# 

**Published:** 1896-08-22

**Authors:** 


**Notices of Births, Deaths, and Marriages** are inserted in this column at a charge of 2s. 6d. for an announcement not exceeding four lines.

### NOTICE TO CORRESPONDENTS.

All MS., Letters, Books for Review, and other matters intended for Editor should be addressed The Editor, The Lodge, Porchester Quare, London, W.

The Editor cannot undertake to return rejected MS., even when accompanied by stamped directed envelope.

**Notice to Advertisers and Subscribers.**

All Advertisements, Orders for Copies of The Hospital and Business Communications should be addressed to **THE MANAGER, (Not to The Editor)**, The Hospital, 428, Strand, London, W.C.

The Cost of Subscription, post free, is as follows:—Per week, 2½d.; per quarter, 2s. 8d.; per half-year, 5s. 3d.; per annum, 10s. 6d. Foreign:—Per Annum, 15s. 2d.; payable, in all cases, in advance.

Price 5s.

**"Burdett's Hospitals and Charities, 1896."**

Seventh year of publication. Over 1,000 pp. Scarlet cloth, gilt lettered, Price 5s. A most complete guide to British, Colonial, Indian, and American Hospitals, Dispensaries, Nursing and Convalescent Institutions, and Asylums. A few complete sets of the preceding six issues of the "Annual" may still be obtained on application to the Publishers, The Scientific Press (Limited), 428, Strand, London, W.C.

### SCRAPS AND GLEANINGS.

Bedford Hospital Saturday has produced £446 this year.

The total collection on Hospital Saturday at Guildford amounted this year to £175, as compared with £156 last year.

The report of the Hull Homœopathic Dispensary for the past two years states that 1,069 cases have been treated during that period.

The average stay of a patient at the Belfast Hospital for Skin Diseases during the past year has been seven weeks. 927 patients were treated.

The annual house-to-house collection in aid of the funds of the Lincoln County Hospital has amounted to £135, a sum rather in excess of that received last year.

A Toronto publisher has furnished and equipped the new hospital at Stornoway, in the Island of Lewis, which has recently been erected by public subscription.

£4,300 has been raised this year by the Bradford Joint Hospital Fund.

The Yeovil Cottage Hospital received £28 as the result of this year's Hospital Sunday parade.

Since the Truro Hospital Saturday Fund was started five years ago the collections have gradually increased, the £48 of the first occasion having given place to £70 last year.

Hospital Sunday in Dumbarton this year realised the sum of £105 3s. 5d., and to that extent the Dumbarton Cottage Hospital will benefit. The amount collected last year was £92 6s. 9d.

Out of a total income of £792 received last year by the Smedley Memorial Hospital at Matlock, £530 was received from patients' payments. The expenditure amounted to £781.

The total receipts of the 1895 collection by the Exeter and District Hospital Saturday Fund were £811, making the sum collected since the foundation of the fund, twelve years ago, £10,148.

The Governors of the Coombe Lying-in Hospital, Dublin, have been requested "to consider the desirability of starting a fund to provide proper accommodation for the staff and students."

At the Ancoats Hospital, Manchester, the number of patients treated during last year was as follows:—In-patients, 1,061; out-patients, 3,713; home cases, 2,205; accidents, 6,316; and dental cases, [illegible]91. Among the legacies received during the year was one of £5,000.

The plans of the new medical pavilion at the Edinburgh Royal Infirmary have been finally agreed to, and the building will contain a bathing department, wards containing 28 beds, convalescent room, nurses' quarters, &c., the cost being estimated at £30,200.

The Home for Epileptics at Maghull treated (including 52 remaining from the previous year) 155 patients during 1895, of whom 30 had been discharged and 8 had died, leaving 117 in the home on December 31st, 1895. The general account shows a deficit of £113, and the building fund a deficiency of £3,700.

We see from the annual report of the Salford Borough Hospital Department that 1,356 patients (659 males and 697 females) were treated at the infectious hospitals during the year 1895, as compared with 1,227 in 1894. Each patient spent on an average 40·1 days in the hospital, and cost £3 17s. 1d., or 13s. 5d. a week.

The Camborne Urban District Council evidently believe in economy, for at a recent meeting it was suggested that the Council invite offers for the tenancy of the infectious hospital, the tenant to be prepared, when there were any patients, to give the necessary attendance to the hospital and nurse, or to quit the premises on 24 hours' notice.

The daily cost of maintenance of patients and staff at the Devizes Cottage Hospital during 1895-6, excluding medicine and establishment charges, is given at 1s. 7d. in the annual report, or including those charges, 3s. 10¾d. 108 patients were admitted, the daily average number of beds occupied being 8·5. Receipts amounted to £690, and expenditure to £838.

The report of the treasurer of the Arbroath Infirmary shows that the income for the year ended July 4th, 1896, amounted to £783, and the ordinary expenditure to £766. The Convalescent Home account shows an income of £143, and an expenditure of £225, the deficiency being met by the infirmary. One hundred and ninety-two in-patients were admitted, and 268 out-patients treated.

A difficulty has arisen between the West Bromwich Hospital Saturday Committee and the managers of the District Hospital with reference to the practice of placing the wages earned by working men received into the institution upon the tickets of admission. The question was raised in consequence of men in receipt of good wages being sent into the hospital for treatment. The committee have passed a resolution requesting that in future the amount of a patient's wages be not stated on his ticket of admission.

The last report of the Orphan Working School concludes with the following: "Your board have to mention one most gratifying incident that occurred in March last. Mrs. Fletcher, the lady who gave the £1,000 in 1894, wrote to the secretary saying that she should like to offer one of her life presentations to H.R.H. Prince Edward of York. This presentation was graciously accepted by his Royal parents, who wrote as follows:—'Will you be pleased to express to Mrs. Fletcher our sense of her kindness, and our gratitude at giving the infant prince a life interest in the institution, which does so much for little children.'"

### The Student of Medicine.

### ST. BARTHOLOMEW'S HOSPITAL.

We learn that a very satisfactory change in the remuneration given to the resident staff has been made by the authorities at St. Bartholomew's Hospital. The house surgeons and house physicians have hitherto only been paid a nominal sum, and have been put to the expense of board. But from October 1st next the junior house physicians and junior house surgeons, who are non-resident, will receive £25 each, and the senior house physicians, and the senior house surgeons, who are resident, will be paid at the rate of £80 a year, this being regarded as the equivalent of board in the College connected with the Hospital. The resident midwifery assistant, the extra midwifery assistant, and the ophthalmic house surgeon will each receive a salary of £80 a year, and the senior and junior resident anæsthetists one of £120 and £100 a year respectively. The substantial increases of salary involve a large additional charge upon the hospital funds, but we are sure that they will give rise to much satisfaction amongst the large number of students in the Medical School.

### APPOINTMENTS.

J. A. Atkinson, M.R.C.S., L.R.C.P.Lond., appointed Medical Officer to the Knaresborough Union Workhouse. D. M. Beddoe, F.R.C.S., appointed House Surgeon to the Newport and Monmouthshire Hospital. E. Bellis, L.R.C.P.I., L.R.C.S.I., appointed Surgeon to the Employés on the West London Central "Electric" Railway Sections, 1, 2, and 3. Alexander Blayney, M.A., M.B.R.U.I., appointed Assistant Surgeon to the Mater Misericordiæ Hospital, Dublin. R. E. Burges, M.D., appointed Medical Officer of Health to the Hoole Urban District Council. H. J. Clark, M.R.C.S., L.R.C.P., appointed Clinical Assistant to the Chelsea Hospital for Women. Martin Dempsey, B.A., M.D.R.U.I., M.R.C.P., appointed Assistant Physician to the Mater Misericordiæ Hospital, Dublin. A. T. Dochard, M.B., C.M.Edin., appointed Medical Officer for the Whitefield District of the Bury Union. Percy Edmunds, M.B., B.Sc.Lond., appointed Medical Officer under the London County Council to No. 7 District (Fire Brigade, &c.) A. R. Friel, M.A., M.D., appointed Clinical Assistant to the Chelsea Hospital for Women. G. F. S. Genge, L.R.C.P.Lond., M.R.C.S.Eng., appointed House Physician to the Westminster Hospital. Dr. C. Grant, appointed Medical Officer for the Priors Marston District of the Southam Union. John Hoyle, M.B., C.M., appointed Medical Officer and Public Vaccinator for the Ripponden District of the Halifax Union. C. Lamplough, M.R.C.S., L.R.C.P., appointed Resident Medical Officer to the City of London Hospital for Diseases of the Chest, Victoria Park. G. Balfour Marshall, M.D., C.M.Edin., F.F.P.S.Glas., appointed Gynæcologist to the Glasgow Royal Infirmary. John O'Donnell, M.B., B.Ch.R.U.I., appointed Medical Registrar to the Mater Misericordiæ Hospital, Dublin. Dr. William Stewart, appointed Medical Officer of Health for Burgh of Gourock. J. Lynn Thomas, F.R.C.S., appointed Consulting Surgeon to the Cardiff Provident Dispensary. C. F. Weinman, M.B., C.M. appointed Clinical Assistant to the Chelsea Hospital for Women. Dr. J. H. Wilson, appointed Medical Officer of Health to the Wigan Rural District Council. T. M. Clayton, M.B., B.S.Durh., appointed Medical Officer of Health to the Felling Urban District Council.

### PASS LISTS.

### University of Edinburgh.

First Professional Examination.—The following is the official list of candidates who have completed this examination: R. Affleck, N. Ahmed, H. Alexander, C. M. Anderson, J. Anderson, W. L. G. Anderson, H. E. Arbuckle, B. S. H. Aylward, M. C. Beatty, J. H. Bell, L. N. Biggs, A. B. Black, O. C. Bradley, W. Brown, G. F. Buist, R. A. Cameron, J. P. Campbell, G. S. Carmichael (with distinction), Lilian M. Chesney, A. D. S. Cooke, G. G. Cumming, Jane E. Dallas, G. C. V. K. Davidson, S. L. Dawkins, J. Duncan, W. Eadie, S. R. Ellis, J. S. Enslin, D. Ewart, W. Ewart, A. Fleming, C. Fraser, W. E. Frost, A. W. Fuller, J. C. Gilchrist, N. Glegg, S. E. Goggin, St. L. H. Gribben, P. S. Haldane, G. H. Hanna (with distinction), F. Hardie, N. E. Harding, W. E. Herbert, D. Heron, Eleanor Hodson, D. B. B. Hughes, W. T. James, E. B. Jamieson, J. G. S. Jamieson, M. E. Jones, J. C. Kennedy, F. K. Kerr, S. M. de Kock, L. S. Lessing, G. H. Lewis, P. N. M. Macdonald, J. G. M'Dougall, Sara L. M'Elderry, W. M'Farlane, W. E. M'Farlane, J. G. MacKenna, D. S. MacKnight, P. A. Maclagan, Harriet J. C. Maclaren, A. M'Nab, G. W. P. Maitland, A. Martin, K. D. Melville, E. G. D. Menzies, R. M. Mitchell, J. Munro, A. W. Nankervis, S. Neumark, Caroline E. O'Connor, J. C. Parker, W. Paterson, N. Patterson, P. Pattison, C. B. Paul, Mary C. Pepper, E. J. Porteous, J. B. Primmer, J. R. Prytherch, S. Rattray, L. C. P. Ritchie, D. J. Roberts, J. K. A. Robertson, J. Ross, W. C. Ross, A. L. Roxburgh, J. Scobie, J. B. Scott, W. S. Scott, S. R. Sibbald, H. O. Smith, C. B. Snow, S. Southall, A. D. Spence, A. J. Spiganovicz, Eleanor R. Sproull, T. G. Stewart, E. Steyn, M. A. Swan (with distinction), Emily C. Thomson, J. W. N. Thomson, J. H. Thornley, A. Trotter, A. Tweedie, K. H. H. Uffmann, Isabel B. Venters, N. N. Wade, A. G. Watson, A. S. Watson, N. S. Wells, G. D. Whyte, F. M. Wigg, A. J. Williamson, A. W. Wilson, G. Wilson, A. M. Wood, and G. Wright.

### Royal College of Surgeons, Edinburgh.

The following gentlemen, having passed the requisite examinations, were admitted Fellows of the College on the 30th ult.: J. A. H. White, M.R.C.S.Eng., Nechells, Birmingham; T. J. Henry, L.R.C.S.E., Sydney; J. Allison, L.R.C.S.E., Fuller House, Kettering, Northamptonshire; J. F. C. Hossack, L.R.C.S.E., Cape Town; G. W. F. Macnaughton, M.B.C.M.Edin., 15, Murchiston Terrace, Edinburgh; and C. Horrocks, L.R.C.S.E., 55, Queen Mary Avenue, Crosshill, Glasgow; and E. G. Horder, L.R.C.S E., 18, Baskerville Road, Wandsorth Common, London, S.W., was admitted a Fellow without examination.

